# Executive control and felt concentrative engagement following intensive meditation training

**DOI:** 10.3389/fnhum.2013.00566

**Published:** 2013-09-18

**Authors:** Anthony P. Zanesco, Brandon G. King, Katherine A. MacLean, Clifford D. Saron

**Affiliations:** ^1^Department of Psychology, University of CaliforniaDavis, CA, USA; ^2^Center for Mind and Brain, University of CaliforniaDavis, CA, USA; ^3^Department of Psychiatry and Behavioral Sciences, Johns Hopkins University School of MedicineBaltimore, MA, USA; ^4^The M.I.N.D. Institute, UC Davis Medical CenterSacramento, CA, USA

**Keywords:** executive control, meditation, response inhibition, sustained attention, task engagement

## Abstract

Various forms of mental training have been shown to improve performance on cognitively demanding tasks. Individuals trained in meditative practices, for example, show generalized improvements on a variety of tasks assessing attentional performance. A central claim of this training, derived from contemplative traditions, posits that improved attentional performance is accompanied by subjective increases in the stability and clarity of concentrative engagement with one's object of focus, as well as reductions in felt cognitive effort as expertise develops. However, despite frequent claims of mental stability following training, the phenomenological correlates of meditation-related attentional improvements have yet to be characterized. In a longitudinal study, we assessed changes in executive control (performance on a 32-min response inhibition task) and retrospective reports of task engagement (concentration, motivation, and effort) following one month of intensive, daily Vipassana meditation training. Compared to matched controls, training participants exhibited improvements in response inhibition accuracy and reductions in reaction time variability. The training group also reported increases in concentration, but not effort or motivation, during task performance. Critically, increases in concentration predicted improvements in reaction time variability, suggesting a link between the experience of concentrative engagement and ongoing fluctuations in attentional stability. By incorporating experiential measures of task performance, the present study corroborates phenomenological accounts of stable, clear attentional engagement with the object of meditative focus following extensive training. These results provide initial evidence that meditation-related changes in felt experience accompany improvements in adaptive, goal-directed behavior, and that such shifts may reflect accurate awareness of measurable changes in performance.

Goal-directed behaviors requiring sustained concentration are ubiquitous in daily life. As a consequence, the ability to voluntarily control attention is essential for promoting academic and professional success, maintaining mental and physical health, and building adaptive interpersonal skills (Tangney et al., 2004). But there are limits on the overall capacity to direct and control attentional resources (Kaplan and Berman, 2010). Perhaps unsurprisingly, individuals commonly find sustaining their concentration during simple tasks to be stressful and effortful (Warm et al., 2008; Langner and Eickhoff, 2013), and momentary lapses can disrupt the stability of attention as the mind drifts on and off task over time (Weissman et al., 2006). Furthermore, individuals are often unaware that their attention has lapsed at all (Smallwood and Schooler, 2006). There is increasing evidence suggesting that directed mental training, including meditation, may serve as one potential method to attenuate deficits in attentional stability (Slagter et al., 2011; Mrazek et al., 2013). Although these studies provide evidence that meditation training may impact neural and behavioral markers of attention and executive control (Hölzel et al., 2011), the extent to which observed improvements are accompanied by corresponding changes in phenomenological aspects of attention is unknown. In the present study, we aim to characterize training-related changes in phenomenal awareness that accompany improvements in sustained, goal-directed attention following intensive meditative practice.

The fluctuating nature of attention has long been acknowledged by several Buddhist contemplative traditions (Wallace, 1999, 2006). These contemplative traditions have developed complex mental training techniques for cultivating stable attention, developing introspective and meta-cognitive abilities, and increasing one's capacity for behavioral and emotional regulation (Lutz et al., 2008). Attention training through meditative practice can thus be conceived as a method for developing central attentional resources for the adaptive regulation of cognition and behavior. During meditative practice, practitioners may employ specific *focused-attention* techniques to selectively maintain attention on an object of concentration, typically the sensations of the breath and body, while monitoring the quality of ongoing awareness (e.g., clear or dull, focused or distracted). Other *monitoring* techniques involve awareness, introspection, and discriminative analysis of the contents of phenomenological experience (e.g., discerning bare sensations from associated evaluations and judgments). Together, these focused-attention and monitoring techniques comprise the basic methods for training in *Vipassana* meditation (Goldstein, 1976; Goldstein and Kornfield, 2001), from which a number of contemplative-based therapies are derived (e.g., Mindfulness-Based Stress Reduction; Grossman et al., 2004; Williams and Kabat-Zinn, 2011).

In line with the notion that meditative training may support generalized improvements in executive control (Slagter et al., 2011), recent longitudinal studies suggest that the ability to coordinate one's attention and behavior in response to task demands may be improved through intensive practice of meditation (Jha et al., 2007; MacLean et al., 2010; Sahdra et al., 2011; Allen et al., 2012; Moore et al., 2012). For example, compared to wait-list controls, meditators who practiced 3 months of focused-attention meditation (*Shamatha*) demonstrated improved performance accuracy in sustained-attention tasks requiring perceptual discrimination of rare targets (MacLean et al., 2010) and were better able to inhibit habitual pre-potent responses (Sahdra et al., 2011). These findings indicate improvements in two constituent processes that underlie human executive control: (1) the maintenance of attentional or perceptual resources over extended periods of time, as indicated by a moderation of the rate of decline in perceptual sensitivity to target stimuli (Parasuraman, 1979; Nuechterlein et al., 1983; See et al., 1995), and (2) the ability to withhold inappropriate pre-potent response tendencies in tasks requiring behavioral inhibition to rare targets (Robertson et al., 1997; Ridderinkhof et al., 2004). Such findings are consistent with a larger body of evidence suggesting that intensive meditation training positively impacts component processes which contribute to poor performance in tasks requiring sustained executive control (Slagter et al., 2011).

The maintenance of goal-directed attention over time and the inhibition of task-inappropriate behavioral responses are thought to involve the coordinated effort of a network of brain regions within frontal and parietal cortices (Miller and Cohen, 2001; Corbetta and Shulman, 2002; Peterson and Posner, 2012; Langner and Eickhoff, 2013). Together, these component processes place considerable processing demands on attentional systems when executive control must be maintained over time (Parasuraman, 1979; Nuechterlein et al., 1983; See et al., 1995). This sustained attentional demand leads to a decline in performance known as the vigilance decrement, which is thought to reflect the depletion of information processing resources that cannot be immediately replenished under the constraints of the current task demands (Warm et al., 2008; Kaplan and Berman, 2010).

Ongoing fluctuations in attention on a moment-to-moment basis may also impact the maintenance of goal-directed attention over time. This notion is supported by an emerging consensus from behavioral and neurophysiological studies that have linked changes in functional connectivity between brain regions underlying attentional control and sensory processing to lapses of attention, as reflected in variability of reaction times (RT) during ongoing task performance (Manly et al., 2000; West et al., 2002; Bellgrove et al., 2004; Weissman et al., 2006, 2009; Kelly et al., 2008; Prado and Weissman, 2011; Prado et al., 2011). Task-unrelated cognitive processing (e.g., mind-wandering; Smallwood and Schooler, 2006) may also underlie attentional variability, as recent studies suggest an association between response time variability and instances of task unrelated thought (Cheyne et al., 2009; Mrazek et al., 2012; Seli et al., 2013). Taken together, the available evidence suggests that ongoing variations in attentional state may contribute significantly to observed behavioral variability when goal-directed attention is maintained over time.

Recent studies pairing subjective measures of attentional engagement to behavioral and physiological markers of lapses in attention have contributed to our understanding of ongoing fluctuations in performance and sensory processing (Smallwood et al., 2004; Christoff et al., 2009; Kam et al., 2010; Macdonald et al., 2011). By probing whether participants were in a focused (on-task) or unfocused (off-task) state, Kam et al. (2010) observed attenuated modulation of visual event-related potentials on trials preceding reports of unfocused states. Furthermore, Macdonald et al. (2011) observed that trial-by-trial ratings of participants' depth of focus (more vs. less focused) predicted target discrimination and were negatively related to pre-stimulus alpha oscillatory power, an electrophysiological marker of attention commonly implicated in stimulus detection. In line with these findings, Lutz et al. (2002) previously demonstrated that verbal descriptions of participants' preparedness to perceive 3D “popouts” in random dot stereograms were associated with increased bilateral synchronization of frontal EEG and improved behavioral performance. By quantifying phenomenological aspects of attention and awareness, these studies illustrate how subjective indices of attention may be used to clarify the cognitive and neural processes that contribute to overall performance outcomes.

Efforts to understand the experiential correlates of cognition may benefit from the investigation of mental training regimens incorporating meditative introspection. Introspective monitoring techniques form a core component of training in Vipsassana meditation, which may facilitate more accurate reporting of subjective mental states than would likely be obtained from individuals untrained in the observation of internal phenomena (Varela, 1996; Lutz and Thompson, 2003). Though results have been mixed (Nielsen and Kaszniak, 2006; Khalsa et al., 2008), there is some evidence supporting the efficacy of these techniques in facilitating introspective accuracy (Sze et al., 2010; Fox et al., 2012). For instance, Fox et al. (2012) reported that meditative experience is related to increased accuracy between self-reported, neural, and behavioral markers of tactile sensitivity. Further, using a tactile detection task, Mirams et al. (2013) observed fewer tactile misperceptions near participants' individual sensory threshold after a 6-day brief intervention of body-scan mindfulness meditation. There is also evidence that meditation training moderates dynamic activation in primary sensory cortices during attentional orienting to tactile stimuli, suggesting a role for the modulation of alpha-band oscillatory activity in processing and filtering sensory information (Kerr et al., 2011, 2013). Thus, meditation practice may promote meta-cognitive and interoceptive capacities that aid practitioners in observing and describing internal mental states and experiences. With increased experience, these reports should more closely mirror processes inferred from externally observable measures. In turn, increased meta-cognitive awareness of attentional states may allow practitioners to better regulate their performance by recognizing and disengaging from distractions and endogenously moderating the stability of their ongoing attention.

A central claim of contemplative training posits that improved attentional performance is accompanied by subjective increases in the stability and clarity of concentrative engagement with the object of meditative focus (Wallace, 1999, 2006). However, there is little direct evidence detailing the potential correspondence between states of felt concentration and improvements in executive control and attentional stability. Despite contemplative (Goldstein, 1976; Wallace, 1999) and psychological (Mrazek et al., 2012) accounts suggesting that increases in experiential concentration may parallel reductions in unwanted and intrusive thoughts, feelings, and sensations, self-reported concentrative engagement has not been directly linked to observed performance outcomes. Contemplative accounts also suggest that less cognitive effort should be required for directing and maintaining attention as expertise develops (Wallace, 1999). In line with this conception, several researchers have interpreted patterns of neural activity during attentional tasks (Lutz et al., 2009) and meditative states (Brefczynski-Lewis et al., 2007; Saggar et al., 2012) as reflecting decreased task-related effort and demand following meditative training. These studies, however, did not attempt to directly assess task demand or effort.

In addition to receiving instruction in specific attentional practices, meditation practitioners are also encouraged to cultivate an enduring motivation to engage with the teachings, techniques, and principles of contemplative practice globally (Goldstein, 1976; Wallace, 1999; Goldstein and Kornfield, 2001; Wallace and Shapiro, 2006). Although these conative factors likely promote effective training, they may also contribute to differences in motivation between experimental conditions, which may confound interpretations of attentional improvements (Jensen et al., 2012). No study of intensive contemplative training has yet addressed this concern by assessing performance motivation. Behavioral improvements observed following meditation training interventions may thus reflect changes in a number of underlying factors, including both attention-specific processes (e.g., endogenous focus and concentration) as well as motivational processes. The incorporation of first-person information about an individual's attentional and motivational state in studies of meditation may help clarify the relative impact of these factors on training outcomes. Taken together, the examination of these aspects of task engagement—concentration, effort, and motivation—may prove useful in clarifying the experiential consequences of directed mental training.

In the present longitudinal study, participants completed a sustained response inhibition task (RIT) (Sahdra et al., 2011) and reported on mental states related to task engagement, a construct encompassing felt concentration, effort, and motivation, before (*pre-test*) and after (*post-test*) an intensive 1-month Vipassana (Insight Meditation) retreat. A group of matched control participants completed identical longitudinal assessments but did not undergo training. Our first aim was to assess the effects of Vipassana meditation training on both response inhibition accuracy and reaction time variability, measured within the same experimental paradigm. Our second aim was to examine changes in task engagement as a result of training. Our third aim was to examine the contribution of potential training-related changes in subjective task experience to improvements in both executive control and attentional stability.

## Methods

### Participants

Training participants underwent a 1-month intensive residential meditation retreat held at Spirit Rock Meditation Center (SRMC) in Woodacre, California. Twenty-eight self-selected individuals were assessed at the beginning and end of the retreat. A comparison group of 27 control participants (matched on demographic variables and estimated lifetime and daily meditation experience; see Table 1 for final participant sample) were recruited from SRMC community meditation classes and were tested before and after an interval of ~1 month (*M* = 27.65 days, *SD* = 3.51 days) onsite at SRMC. Control participants had previous experience with meditation but did not undergo intensive training during the time between assessments and had not completed any retreats up to 4 weeks prior to beginning the study. All study details were approved by the University of California, Davis institutional review board. Participants gave informed consent at the first study assessment and were debriefed at the end of the second assessment. Participants were compensated $120 for their participation.

**Table 1:** **Group matching on demographic and experience variables**.

**Measure**	**Control group**	**Training group**	**All participants**	***t*-value (*df*)**	***p*-value**
Age (years)	54.70 (23–72)	49.62 (25–70)	52	1.345 (47)	0.19
Sex	5 Male, 19 Female	8 Male, 18 Female	13 Male, 37 Female	−	−
Education	4.74 (2–6)	5.08 (4–6)	4.92	0.994 (47)	0.33
Income	7.38 (1–11)	8.23 (1–11)	7.82	0.905 (48)	0.37
Mean meditation (min/day)	31.72 (6–120)	41.52 (0–320)	36.92	0.733 (47)	0.47
Lifetime meditation (hours)	1767.46 (76–9265)	3311.52 (165–15000)	2556.64	1.753 (43)	0.09
Years of experience	9.91 (1–30)	13.67 (3–39)	11.91	1.410 (47)	0.17

Mean values and ranges are provided for demographic and meditation experience variables for the final behavioral sample (control n = 24, training n = 26). Between-group t-tests revealed no significant differences on reported variables (all ps > 0.05) at initial assessment. Education was scored on the following scale: 1, less than high school diploma; 2, high school diploma; 3, some college; 4, college degree; 5, some graduate study; 6, graduate degree. Total annual household income was reported on the following scale: 1, $10,000 or less; 2, $10,001–20,000; 3, $20,001–30,000; 4, $30,001–40,000; 5, $40,001–50,000; 6, $50,001–60,000; 7, $60,001–70,000; 8, $70,001–80,000; 9, $80,001–90,000; 10, $90,001–100,000; 11, More than $100,000. Estimated meditation experience variables included average daily minutes of formal meditation practice during the past month, total number of lifetime hours of formal meditation practice, and number of years practicing meditation.

### Meditation training

Training involved a collection of techniques known as Vipassana meditation, drawn from the Theravadan Buddhist tradition (Goldstein and Kornfield, 2001). Instruction during the retreat was provided by multiple experienced SRMC teachers. Meditation techniques involved the repeated application of attention to the physical sensations of the breath, the observation and identification of sensations, thoughts, desires, intentions, and emotions, and the meta-cognitive monitoring of the quality of attention and diverse mental states. Furthermore, participants engaged in a number of aspirational and emotion-generative meditation practices emphasizing the cultivation of compassion and loving-kindness (Salzberg, 2002) to supplement the primary training. Participants maintained silence during the duration of the retreat and typically attended thirteen 45-min meditation sessions each day (seven sitting sessions and six walking sessions).

### Testing procedures

Training group participants were tested on the morning of the first and last day of the retreat. Testing sessions took place in participants' individual dormitory rooms. Each participant was provided with a box containing an IBM T-40 ThinkPad laptop equipped with Presentation software (Neurobehavioral Systems, http://www.neurobs.com) to control stimulus delivery and record behavioral responses, as well as materials and instructions for assembling the testing station. Instructions were included for setting dim ambient lighting (e.g., blocking window light and using a low-wattage lamp) and maintaining a viewing distance of 57-cm from the computer screen. Control group participants underwent identical testing procedures in the same dormitories. At each testing session, participants completed the RIT immediately followed by retrospective questionnaire measures of task engagement. The RIT was the second of six behavioral tasks completed at each assessment.

### Response inhibition task

#### Threshold

Participants first completed an ~10-min threshold procedure to calibrate task difficulty for each individual in order to equate task demand across participants. Participants maintained eye gaze fixation on a small dot at the center of the screen while they viewed single gray vertical lines appear one at a time against a black background. Each stimulus was presented for 150 ms. The inter-stimulus interval (ISI) varied randomly but was constrained to have a mean of 1850 ms and a range not exceeding 1550–2150 ms. A variable ISI was used to minimize the potential performance benefit gained from a predictable stimulus, thereby increasing overall task demand (MacLean et al., 2009). Participants responded as quickly and accurately as possible with the left mouse button (right index finger) to frequent long lines (70% of stimuli) while withholding responses to rare short lines (30% of stimuli) and received sound feedback through headphones (Sony MDR-V150). Auditory feedback consisted of a *ding* when participants correctly withheld their response to the short line target and a *woosh* when participants incorrectly withheld their response, or failed to respond, to a long line non-target. The length of the short line was adjusted according to Parameter Estimation through Sequential Testing (PEST) until converging on an overall accuracy of 75% (see details in MacLean et al., 2009).

#### RIT

Next, participants completed the 32-min RIT (960 trials in total) with the short target line length set to each participant's individual threshold. At both assessments, the length of the short line was set to the participant's pre-test threshold in order to equate task parameters across assessments. Stimulus and response parameters for the RIT were the same as for the threshold procedure, except that target lines occurred less frequently (10% of all stimuli totaling 96 target lines), the length of the target line remained the same throughout the task, and there was no sound feedback.

#### Analysis

Response inhibition accuracy was quantified using the non-parametric index of perceptual sensitivity, *A*′. When hit rate is greater than false alarm rate, *A*′ is calculated as $A^{\prime }=0.5+\frac{\left(H\;-\;F\right)\left(1\;+\;H\;-\;F\right)}{4H(1\;-\;F)}$; when hit rate is less than false alarm rate, *A*′ is calculated as $A^{\prime }=0.5-\frac{\left(F\;-\;H\;\right)\left(1\;+\;F\;-\;H\right)}{4F(1\;-\;H)}$ (see Stanislaw and Todorov, 1999; hits were defined as correct inhibitions to targets and false alarms were defined as incorrect inhibitions to non-targets). This index commonly ranges from 0.5 to 1, with the former value reflecting chance performance and the latter value perfect performance. RT variability for each participant was quantified as the reaction time coefficient of variability (RT CV = standard deviation RT/mean RT) for non-target trials. For each participant at each assessment, *A*′ and RT CV were calculated for the overall task and for each of eight contiguous trial blocks. Each block contained 120 trials and lasted 4 min.

We analyzed training-related changes in *A*′ and RT CV using multi-level models with SAS PROC MIXED version 9.3 in order to examine linear trajectories of growth across the eight blocks of the RIT. Fixed effects in these models are interpreted as regression coefficients (i.e., a parameter estimate represents the expected difference in the dependent variable given a one-unit increase in the independent variable while holding the other variables constant). For all analyses, independent variables representing group (control = 0, training = 1) and assessment (pre-assessment = 0, post-assessment = 1) were treated as dummy variables. Block was centered to the first 4-min block (block 1 = 0) and this parameter represents the linear trajectory (slope) of performance across each 4-min segment of the RIT.

### Self-report measures of task engagement

Immediately following the RIT, participants completed two self-report measures from the Dundee Stress State Questionnaire (DSSQ; Matthews et al., 2002) to retrospectively assess motivation (14 items; e.g., “I wanted to succeed on the task,” “I felt apathetic about my performance”) and concentration (7-item sub-scale from the 30-item thinking style questionnaire; e.g., “I found it hard to maintain my concentration for more than a short time,” “My mind wandered a great deal”) experienced during task performance. Each item was rated from 0 (“not at all”) to 4 (“extremely”), reverse scored items were corrected, and items were summed to obtain scale scores. Thus, larger scores indicate high levels of concentration or motivation. Cronbach's alpha reliability coefficients indicated acceptable levels of consistency among items in the concentration scale (α = 0.79 for pre- and α = 0.85 for post-test), and marginally acceptable levels for the motivation scale (α = 0.65 for pre- and α = 0.62 for post-test). In addition, participants were asked to report: the amount of mental, physical, and temporal demand experienced during task performance; effort devoted to task performance; the degree to which they achieved their performance goals; and the perceived frustration induced by the task by rating six independent items taken from the NASA-TLX (Hart and Staveland, 1988) on a scale from 0 (“low”) to 10 (“high”). Following Matthews et al. (2002), these six items were averaged to obtain an overall measure of task effort and demand.

## Results

There were no significant differences between groups on demographic or meditation-experience variables at pre-test (see Table 1). Two participants (1 training) were excluded from analyses because performance was below or near chance at one of the two assessments (>3 *SD* lower than mean *A*′ of the sample, *M* = 0.895, *SD* = 0.072), strongly suggesting these participants did not comply with or understand the task instructions, resulting in an uninterpretable change in performance across assessments. One additional participant (control) was excluded due to near chance level performance in overall *A*′ at both assessments (>3 *SD* lower than mean *A*′). Finally, two participants were excluded due to an interruption of the testing session (control) and explicit failure to comply with task instructions (training). Thus, the final behavioral sample included 26 training and 24 comparison group participants. Among these participants, individuals with incomplete questionnaire data were excluded from respective analyses on concentration (training: *n* = 23; control: *n* = 24), effort (training: *n* = 23; control: *n* = 24), and motivation (training: *n* = 22; control: *n* = 23).

### RIT performance

#### Threshold

The purpose of the threshold procedure was to maintain constant task difficulty across participants. Although we did not anticipate training-specific changes in threshold, we tested for possible effects of group (training vs. control) and assessment (pre-test vs. post-test) using repeated measures analysis of variance (ANOVA). We found a main effect of assessment [*F*_(1, 48)_ = 48.850, *p* < 0.001, η^2^_*p*_ = 0.504], no significant effect of group [*F*_(1, 48)_ =1.948, *p* = 0.169], and no significant interaction between assessment and group [*F*_(1, 48)_ = 0.009, *p* = 0.924]. These results are consistent with general practice effects and suggest that the groups had comparable threshold values across assessments (pre control target visual angle *M* = 3.51°, *SD* = 0.66°; post control *M* = 3.94°, *SD* = 0.57°; pre training *M* = 3.72°, *SD* = 0.63°; post training *M* = 4.17°, *SD* = 0.48°).

#### Mean reaction time

In order to rule out response time slowing as a factor influencing performance improvements we examined whether groups differed in their overall mean reaction time across all non-target trials. A repeated measures ANOVA on the within-subjects effects of assessment (pre-assessment and post-assessment), the between subjects effects of group (control and training), and their interaction, demonstrated no significant main effects for group [*F*_(1, 48)_ = 0.611, *p* = 0.438], assessment [*F*_(1, 48)_ = 0.013, *p* = 0.911], or their interaction [*F*_(1, 48)_ = 0.368, *p* = 0.547]. These analyses show that the groups did not systematically differ in overall RT for the long-line stimuli (i.e., non-target trials) across assessments (pre control *M* = 531.52 ms, *SD* = 110.48 ms; post control *M* = 525.89 ms, *SD* = 93.02 ms; pre training *M* = 501.70 ms, *SD* = 133.25 ms; post training *M* = 509.87 ms, *SD* = 102.29 ms).

#### Accuracy

Multi-level models were used to examine changes in response inhibition accuracy (*A*′) over the 32-min task as a function of the fixed effects of task block (centered to the first 4-min block), assessment (centered to the pre-assessment), and group (centered to the control group). We included random effects on the intercept and slope across blocks to allow for individual differences in initial *A*′ and the slope of *A*′ across blocks of the task. We first tested a model including the effects of block, group, and assessment.^1^ This model predicted a significant effect of block (β = −0.006, *p* < 0.001), indicating a decline in *A*′ over the course of the task. This parameter (β = −0.006) reflects the amount of linear decline observed in mean *A*′ across each of the eight contiguous 4-min blocks of the task. A significant effect of assessment was also found (β = 0.048, *p* < 0.001), suggesting that participants improved in overall perceptual sensitivity at the second assessment. There was no effect of group (β = 0.013, *p* = 0.387).

**Figure 1:**
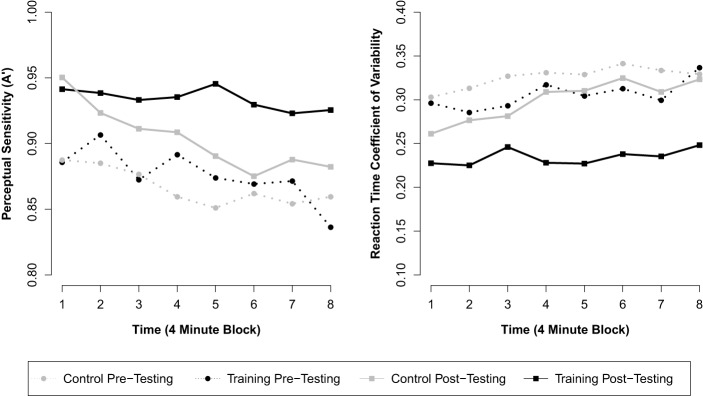
**Mean response inhibition accuracy and reaction time variability across eight contiguous 4-min blocks of the RIT by group and assessment**.

Next we included the interaction term between assessment and group to investigate training-related changes in overall *A*′. This model revealed a significant interaction between assessment and group (β = 0.021, *p* = 0.029; see Table 2 for parameter estimates), consistent with our hypothesis that meditation training would improve response inhibition accuracy. This parameter estimate (β = 0.021) reflects the mean increase in *A*′ across assessments for the training group over and above the change observed for control participants. Thus, although a significant effect of assessment was observed for control participants (β = 0.037, *p* < 0.001; pre *M* = 0.867 A', post *M* = 0.904 A'), suggesting probable test-retest effects, the overall change for training participants (β = 0.058, *p* < 0.001; pre *M* = 0.876 A', post *M* = 0.934 A') was significantly greater than for control participants (see Figure 1). A third model including all two-way and three-way interactions between block, assessment, and group revealed a significant three-way interaction (β = 0.009, *p* = 0.039), suggesting that training moderated the decline in performance over blocks of the task. This final model (BIC = −1820), however, did not fit the data better than the second model (BIC = −1826).

**Table 2:** **Parameter estimates from models of RIT performance**.

**Model and parameter**	**Estimate (SE)**	**Test statistic**	**BIC**
**ACCURACY (*A'*)**			**−1826**
**Fixed effects**			
β0–intercept	0.890 (0.012)	76.82^***^	
β1–block	−0.006 (0.001)	3.78^***^	
β2–assessment	0.037 (0.007)	5.22^***^	
β3–group	0.002 (0.016)	0.88	
β4–group × assessment	0.021 (0.009)	2.19^*^	
**Random effects**			
σ^2^_0_ (intercept)	0.002 (0.001)	3.33^***^	
σ_0, 1_ (covariance)	0.001 (0.001)	0.98	
σ^2^_1_ (slope)	0.001 (0.001)	2.45^**^	
σ^2^_e_ (residual variance)	0.005 (0.000)	18.71^***^	
**REACTION TIME VARIABILITY (RT CV)**			**−2051**
**Fixed effects**			
β0–intercept	0.304 (0.015)	19.82^***^	
β1–block	0.005 (0.001)	3.83^***^	
β2–assessment	−0.026 (0.006)	4.46^***^	
β3–group	−0.020 (0.023)	0.89	
β4–group × assessment	−0.045 (0.008)	5.44^***^	
**Random effects**			
σ^2^_0_ (intercept)	0.005 (0.001)	4.35^***^	
σ_0, 1_ (covariance)	0.001 (0.001)	1.25	
σ^2^_1_ (slope)	0.001 (0.001)	2.39^**^	
σ^2^_e_ (residual variance)	0.003 (0.000)	18.71^***^	

Full maximum likelihood estimates are reported for the best fitting models of change in A' and RT CV across the fixed effects of block (first 4-min block = 0), group (control = 0, training = 1), and assessment (pre-assessment = 0, post-assessment = 1) in the RIT (n = 50). Standard errors are reported in parentheses. Test statistics are reported as t-values for fixed effects estimates and z-values for random effects estimates. Bayesian information criterion (BIC) is reported with lower values (more negative) indicating a better model fit.

* p < 0.05;

** p < 0.01;

*** p < 0.001.

#### Reaction time variability

A similar analysis was used to assess changes in reaction time variability (RT CV) over the 32-min RIT. The first model demonstrated a significant effect of block (β = 0.005, *p* < 0.001), such that RT CV increased over the course of the 32-min task. In addition, a significant effect of assessment was found (β = −0.050, *p* < 0.001), but there was no effect of group (β = −0.033, *p* = 0.111). After including the interaction between assessment and group in the model, we found a significant interaction between assessment and group (β = −0.045, *p* < 0.001; see Table 2 for parameter estimates and test statistics), consistent with our hypothesis that meditation training would reduce fluctuations in response time. This parameter (β = −0.045) reflects the mean decrease in RT CV across assessments for the training group over and above the change observed for the control group. Thus, although a significant effect of assessment was observed for control participants (β = −0.026, *p* < 0.001; pre *M* = 0.326 RT CV, post *M* = 0.299 RT CV), the overall decrease for training participants (β = −0.071, *p* < 0.001; pre *M* = 0.306 RT CV, post *M* = 0.234 RT CV) was significantly greater (see Figure 1). As in the analysis of *A*′, a third model including all two-way and three-way interactions between block, assessment, and group revealed a significant three-way interaction (β = −0.007, *p* = 0.037), suggesting that meditation training moderated the increase in variability over blocks of the task. The addition of these variables, however, did not improve model fit (BIC = −2045) over the second model (BIC = −2051).

### Task engagement

Repeated measures ANOVAs were used to test the effects of assessment (pre-test vs. post-test) and group (training vs. control) on each of the three measures of felt task engagement (see Table 3 for descriptive statistics).

**Table 3:** **Means and standard deviations for self-reported task engagement**.

	**Pre-test Mean (SD)**	**Post-test Mean (SD)**
**TRAINING**		
Concentration	20.65 (4.56)	23.65 (2.21)
Effort and Demand	6.02 (1.20)	5.68 (1.35)
Motivation	28.29 (7.16)	28.95 (9.93)
**CONTROL**		
Concentration	20.50 (4.84)	20.29 (5.65)
Effort and Demand	5.92 (0.96)	6.23 (0.91)
Motivation	28.21 (9.09)	26.87 (9.91)

#### Concentration

For self-reported concentration, the ANOVA revealed a main effect of assessment [*F*_(1, 45)_ = 7.151, *p* = 0.010, η ^2^_*p*_ = 0.137], a non-significant effect of group [*F*_(1, 45)_ = 2.102, *p* = 0.154], and a significant interaction between assessment and group [*F*_(1, 45)_ = 9.445, *p* = 0.004, η ^2^_*p*_ = 0.173]. *Post-hoc* comparisons revealed a significant increase from pre- to post-test in the training group [*t*_(22)_ = −4.374, *p* < 0.001, *d* = 0.78], but not the control group [*t*_(23)_ = 0.266, *p* = 0.79]. The training group participants also reported greater concentration at post-test than did controls [*t*_(45)_ = −2.662, *p* = 0.011, *d* = 0.73].

#### Effort and demand

For self-reported effort and demand, the ANOVA revealed no significant effects of assessment [*F*_(1, 45)_ = 1.997, *p* = 0.164], group [*F*_(1, 45)_ = 0.225, *p* = 0.637], or their interaction [*F*_(1, 45)_ = 0.289, *p* = 0.594]. Thus, the groups did not differ in their self-reported effort during the RIT at either assessment.

#### Motivation

For self-reported motivation, the ANOVA revealed no significant effects of assessment [*F*_(1, 43)_ = 0.085, *p* = 0.772], group [*F*_(1, 43)_ = 0.197, *p* = 0.660], or their interaction [*F*_(1, 43)_ = 0.726, *p* = 0.399]. Thus, levels of motivation did not differ between groups at either assessment.

### Task engagement as a predictor of RIT performance

Next, we examined the relation between measures of task engagement and measures of performance on the RIT across participants. Individual measures of task engagement (concentration, effort, and motivation) were included as predictors of RIT performance at both pre- and post-assessment in a series of multiple regressions. Predictors were entered simultaneously as a set in order to explore whether overall task engagement was a predictor of RIT performance. We also examined whether each individual predictor uniquely explained variance in measures of RIT performance while controlling for the other measures of task engagement.

In the first multiple regression, we examined whether pre-test task engagement explained a significant amount of variance in pre-test *A*′. As a whole, task engagement (concentration, effort, and motivation) was not a significant predictor of pre-test *A*′ [*R*^2^ = 0.128, *F*_(3, 41)_ = 1.998, *p* = 0.129]. The examination of individual parameters revealed that pre-test concentration was the only significant predictor of pre-test *A*′ in the model (*B* = 0.005, *p* = 0.049, *sr*^2^ = 0.088). Effort (*B* = −0.003, *p* = 0.782, *sr*^2^ = 0.002) and motivation (*B* = 0.001, *p* = 0.546, *sr*^2^ = 0.008) did not uniquely explain any variance in pre-test *A*′ when controlling for the other predictors. Next, we examined whether pre-test task engagement was a significant predictor of pre-test RT CV. Task engagement did not predict a significant amount of variance in RT CV at the first assessment [*R*^2^ = 0.116, *F*_(3, 41)_ = 1.787, *p* = 0.165]. Pre-test concentration was the only parameter of task engagement that approached significance (*B* = −0.006, *p* = 0.072, *sr*^2^ = 0.077), whereas effort (*B* = −0.015, *p* = 0.297, *sr*^2^ = 0.027) and motivation (*B* = −0.001, *p* = 0.625, *sr*^2^ = 0.006) did not.

In the next series of multiple regressions, we examined whether post-test measures of task engagement explained a significant amount of variance in post-test measures of RIT performance. At post-test, the set of task engagement predictors explained a significant amount of variance in post-test *A*′ [*R*^2^ = 0.411, *F*_(3, 41)_ = 9.535, *p* < 0.001]. Concentration, however, was the only significant parameter in the model (*B* = 0.007, *p* < 0.001, *sr*^2^ = 0.237), whereas motivation (*B* = 0.001, *p* = 0.183, *sr*^2^ = 0.026) and effort (*B* = −0.006, *p* = 0.387, *sr*^2^ = 0.011) were not. As with post-test *A*′, we observed that task engagement explained a significant amount of variance in RT CV at post-test [*R*^2^ = 0.443, *F*_(3, 41)_ = 10.890, *p* < 0.001]. Once again, concentration was the only significant individual parameter in the model (*B* = −0.011, *p* < 0.001, *sr*^2^ = 0.273), while motivation (*B* = −0.001, *p* = 0.403, *sr*^2^ = 0.010) and effort (*B* = 0.013, *p* = 0.168, *sr*^2^ = 0.027) were not significant predictors of RT CV.

Concentration was the only measure of task engagement found to be a consistent predictor of performance after controlling for other measures of task engagement across the multiple regression analyses. In another set of regressions, we therefore followed-up these analyses examining the relation between concentration and measures of RIT performance alone. At pre-test, concentration predicted a significant amount of variance in both *A*′ [*R*^2^ = 0.113, *F*_(1, 45)_ = 5.707, *p* = 0.021] and RT CV [*R*^2^ = 0.083, *F*_(1, 45)_ = 4.097, *p* = 0.049]. Similarly, post-test concentration predicted a significant amount of variance in both post-test *A*′ [*R*^2^ = 0.368, *F*_(1, 45)_ = 26.229, *p* < 0.001] and post-test RT CV [*R*^2^ = 0.403, *F*_(1, 45)_ = 30.436, *p* < 0.001]. Thus, across all analyses, individuals who reported more felt concentration demonstrated greater RIT accuracy and lower RT variability. The addition of predictors representing group and the interaction between group and concentration did not add significantly to the explained variance in any of these models, suggesting that the relation between concentration and measures of RIT performance was not moderated by group membership at either assessment.

### Predicting changes in RIT performance from changes in concentrative engagement

Our third aim was to relate changes in task engagement to RIT performance following meditative training. As the results indicated significant training-related increases in concentrative engagement, we used hierarchical multiple regressions to investigate whether increased felt concentration predicted improvements in RIT performance among training group participants. Specifically, we tested the unique variance explained by changes in concentration on mean *A*′ and RT CV for the training group (*n* = 23).

In the first hierarchical multiple regression, post-test *A*′ served as the dependent variable. The first step included pre-test *A*′ and pre-test concentration as predictors in order to account for initial performance and concentration levels prior to training. These predictors explained a significant amount of variance [*R*^2^ = 0.355, *F*_(2, 20)_ = 5.493, *p* = 0.013], in post-test *A*′. In the second step, the addition of post-test concentration did not add significantly to the explained variance of the model [Δ*R*^2^ = 0.027, Δ*F*_(1, 19)_ = 0.831, *p* = 0.374]. This suggests that changes in self-reported concentration did not predict training-related improvements in response inhibition accuracy.

**Figure 2:**
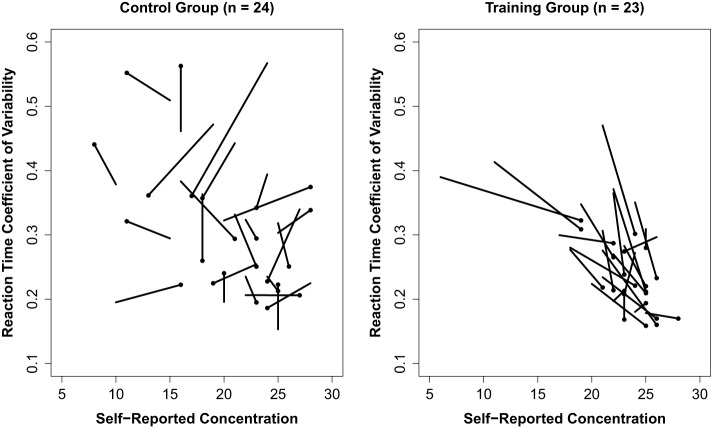
**Plot of change across pre- and post-assessments for reaction time variability and concentration for training and control group participants.** Each vector represents the trajectory from pre- to post-assessment scores for a given individual. Post-assessment scores are represented by a dot. Thus, each vector represents the direction and magnitude of change in these variables for each individual.

A second hierarchical multiple regression was conducted on post-test RT CV using the same analytic strategy. Inclusion of pre-test RT CV and pre-test concentration explained a significant amount of variance in post-test RT CV [*R*^2^ = 0.623, *F*_(2, 20)_ = 16.503, *p* < 0.001]. The addition of post-test concentration to the model explained a significant amount of unique variance in post-test RT CV [Δ*R*^2^ = 0.079, Δ*F*_(1, 19)_ = 5.019, *p* = 0.037], indicating that increases in felt concentration were predictive of reductions in RT CV following training (*B* = −0.009, *p* = 0.037; see Table 4 for parameter estimates). Figure 2 depicts the magnitude and trajectory of changes in concentration and RT CV plotted separately for both training and control groups.

**Table 4:** **Changes in RT CV predicted by changes in felt concentration (dependent variable is RT CV at post-test)**.

**Step 1 (*R*^2^ = 0.623)**	***B***	***SE***	β	***t*-value (*df*)**	***p*-value**
Constant	0.147	0.055	−	2.701 (20)	0.014
RT CV at pre-test	0.435	0.100	0.670	4.364 (20)	0.001
Concentration at pre-test	−0.002	0.002	−0.215	1.398 (20)	0.177
**Step 2 (*R*^2^ = 0.702)**					
Constant	0.320	0.092	−	3.489 (19)	0.002
RT CV at pre-test	0.394	0.093	0.606	4.242 (19)	0.001
Concentration at pre-test	0.001	0.002	0.070	0.369 (19)	0.717
Concentration at post-test	−0.009	0.004	−0.424	2.240 (19)	0.037

## Discussion

The present longitudinal study of intensive Vipassana meditation adds to a growing body of evidence indicating that the capacity for executive control and attentional stability may be improved through directed mental training. We observed training-related improvements in performance accuracy and decreased reaction time variability on a sustained RIT, as well as increases in participant-reported concentrative task engagement. Critically, training-related increases in self-reported concentration predicted reductions in RT variability. This suggests that the experience of clear and unwavering concentration may be a phenomenological correlate of stable attention, reported and felt in aggregate by individuals undergoing introspective meditative training.

Theories of sustained attention propose that the vigilance decrement reflects the consumption of executive resources, which are depleted as attention is maintained over time (Warm et al., 2008; MacLean et al., 2009). Consistent with prior research, we observed a decline in performance over the course of the 32-min RIT. We also observed an increase in reaction time variability as the task progressed. Thus, in addition to decrements in perceptual sensitivity (*A*′), increases in reaction time variability over the course of task performance may reflect an additional feature of resource depletion during sustained response inhibition. This depletion of executive resources over time may lead to increased behavioral variability, as fewer resources are available for maintaining attention on the task set and regulating behavior. Reaction time variability may also result in part from fluctuations in the stability of attention as awareness is drawn to task-unrelated thoughts (Seli et al., 2013). Accordingly, this depletion of attentional resources might impact the ability to resist task disengagement and subsequent mind-wandering by consuming necessary executive resources.

The observed improvements in response inhibition accuracy are consistent with previous findings following intensive, focused-attention (Shamatha) meditation training. Using an identical RIT, Sahdra et al. (2011) reported improvements in perceptual sensitivity (overall *A*′) and sustained performance (slope of *A*′ over task duration) after ~1.5 months of full-time meditation practice. While often articulated as conceptually distinct, focused-attention and monitoring techniques likely engage many of the same attentional and executive processes (Lutz et al., 2008; Slagter et al., 2011). In the present study, both techniques were employed as components of Vipassana training. Thus, our findings of improved attentional stability and response inhibition cannot be attributed solely to the incorporation of either focused-attention or monitoring techniques in the training undertaken by our study participants. Furthermore, training appeared to moderate the typical linear decline in ongoing performance (*A*′ and RT CV) over the duration of the task. Although this result is theoretically important, the statistical models including the interaction of training and performance over blocks did not improve model fit compared to models considering only overall training-related changes. It remains unclear, however, whether these behavioral results are mediated by training-related changes in some other attentional or cognitive process, such as visual working memory capacity which was not measured in the current study.

Although reaction time variability fails to encode the full complexity of ongoing sensorimotor dynamics over the course of an experiment, it nonetheless reflects a useful measure of variability in attention on a trial-to-trial basis. The current findings are consistent with previous studies in which reductions in RT variability were observed following intensive meditation interventions (Lutz et al., 2009; van Vugt and Jha, 2011). These studies, however, did not report evidence for improved performance accuracy alongside reductions in variability. Notably, Lutz et al. (2009) investigated the neural correlates of improvements in attentional stability using an auditory sustained-attention paradigm. They demonstrated reductions in RT variability and enhanced phase consistency of oscillatory neural responses in the theta band following 3 months of Vipassana training. Increased theta phase-locking to stimulus onset over anterior scalp regions predicted the reduction in RT variability. Thus, the ongoing engagement of attention may entrain oscillatory neural responses to task-related sensory input, suggesting a potential neural correlate of observed improvements in attentional stability.

Meditation experience has been linked to increased functional coupling between the posterior cingulate, dorsal anterior cingulate, and dorsolateral prefrontal cortex, which are regions that have been consistently implicated in processes of attention and executive control (Brewer et al., 2011). Similarly, increased connectivity has been observed between bilateral regions of the dorsolateral prefrontal cortex and the right insula in meditation practitioners (Farb et al., 2007; Hasenkamp and Barsalou, 2012). Finally, both cross-sectional (e.g., Lazar et al., 2005) and longitudinal (e.g., Hölzel et al., 2011) studies have demonstrated that Vipassana meditation is associated with increased cortical thickness in prefrontal, anterior cingulate, insular, and somatosensory cortices. These neuroimaging results provide indirect evidence that meditation-related improvements in tasks requiring directed attention and executive control may be supported by cortical plasticity and increased connectivity in regions associated with sensory processing, interoceptive awareness, and the voluntary control and maintenance of attention (Farb et al., 2013; Kerr et al., 2013). Of particular relevance to the study of phenomenological correlates of attentional states, connectivity and cortical plasticity in regions supporting executive and interoceptive processes, such as insular cortex, may support greater subjective awareness of mental states and bodily experiences (Craig, 2009).

The present findings are also consistent with studies of non-meditative interventions demonstrating improvements in executive control in individuals trained to regulate habitual behavior. For example, Muraven (2010) reported improvements in response inhibition following 2 weeks of practiced self-control over common urges in daily life (e.g., avoiding tempting, unhealthy food). Intensive meditation retreats typically involve continuous behavior regulation, such as walking and eating slowly, maintaining meditative posture, and refraining from conversation and eye contact with one's peers. Thus, learning to regulate habitual daily behaviors may facilitate an enhanced capacity to inhibit pre-potent response tendencies, independent of mental training exercises. Other non-specific factors, such as sustained exposure to the natural wilderness setting of the residential retreat center, may have provided additional cognitive benefits (e.g., Berman et al., 2008). One possibility is that attention may be regulated exogenously by the modest stimulation of the natural environments, allowing attentional resources to replenish (Kaplan, 1995; Berman et al., 2008; Kaplan and Berman, 2010). Open, or non-directed, monitoring of internal experiences, as might be practiced during Vipassana meditation, may have similar recuperating effects, as attention is lightly engaged and drawn to the natural internal milieu. An important avenue for future research will be to clarify the contribution of non-specific factors of the meditation-training environment and regimen to the improvement of executive control.

By incorporating experiential measures of task engagement, the present study corroborates contemplative phenomenological accounts of stable, clear concentration while directing attention following extensive training (Goldstein, 1976; Wallace, 1999; Goldstein and Kornfield, 2001). At both assessments and for both groups, the experience of concentrative focus accounted for substantial variance in accuracy and behavioral variability in the sustained RIT. Furthermore, although self-reported concentration related to performance, we observed no group difference in this pattern, contrary to what might be expected given a potential role for meditation training in facilitating the introspection of mental states. We speculate that such a difference might have been observed more readily had we compared participants trained in meditation to those inexperienced with the techniques, consistent with findings from previous cross-sectional studies (Sze et al., 2010; Fox et al., 2012). This pattern, observed across all sample participants, suggests a strong association between subjective feelings of concentration and task performance in the overall sample of experienced meditators. This indicates that mental states of concentration may comprise a potentially important explanatory construct for understanding individual differences in attentional and executive lapses.

Training appeared to increase the quality of concentrative engagement, which in turn predicted improved behavioral stability. Nonetheless, increases in felt concentration did not predict improvements in response inhibition accuracy. Thus, when retrospectively considering aggregate levels of felt concentration, one's sense of moment-by-moment fluctuations in attention over trials may be more phenomenologically accessible than other experiential features of ongoing awareness. In contrast, increases in target discrimination and inhibition of pre-potent responses presumably reflect the more indirect consequences of task attentiveness. Therefore, levels of performance accuracy may serve as a less salient cue when inferring one's attentional state than the ebb and flow of awareness across trials. However, despite training-related differences in subjective and behavioral markers of concentration, there were no training-related changes in the felt experience of mental effort and demand during task performance. Because reports of felt concentration, motivation, and effort were collected retrospectively, after completion of the task, our findings do not speak directly to participants' moment-to-moment experience. It will be interesting to examine whether meditation practitioners are aware of ongoing fluctuations in attentional and motivational states using online reporting and experience sampling techniques, as opposed to aggregated retrospective reports.

It is unclear, however, whether increased introspective accuracy contributes to the observed association between changes in felt concentration and attentional stability. It is possible that training participants' self-reports of concentration at the post-assessment more accurately reflected their measures of performance than did their reports at pre-assessment. In contrast, we interpret our findings as reflective of concomitant increases in phenomenological and behavioral correlates of attentional stability. This interpretation is supported by training group participants reporting higher mean levels of self-reported concentration than controls following training. Furthermore, the strength and direction of the association between concentration and RT variability did not differ between groups at post-assessment. The question of increased introspective accuracy, however, cannot be definitely answered with the current study design. Future studies should determine if training-related improvements in introspective accuracy are found independent of corresponding improvements in performance by including measures not sensitive to training.

There is increasing concern that studies of contemplative training may be susceptible to motivational confounds (Jensen et al., 2012). It is often presumed that self-selected samples are highly motivated to perform well and report personal benefits of training. In particular, practitioners of meditation may hold beliefs about the efficacy of practice that bias their performance by encouraging them to devote more cognitive resources to accomplish performance goals. Although self-selection was a potential confound in the present study, we observed no differences in task-specific motivation and task effort between the training group and the meditation-experience-matched control group. Furthermore, motivation and cognitive demand were not consistent predictors of performance outcomes, challenging the notion that these factors have strong influence on behavioral outcomes among experienced meditators. This is in contrast to cross-sectional studies comparing naïve and novice participants (see Jensen et al., 2012). Our use of demographic and meditation-experience-matched training and control groups may have allowed us to equate levels of motivation, as both groups presumably shared similar convictions and biases regarding the beneficial effects of meditation practice.

The groups were adequately matched on both pre-test performance and overall meditation experience, however, the measures of meditation-experience used here to match participants may not be reliable indicators of participants' lifetime experience. Some participants reported difficulty in attempting to estimate their lifetime meditation experience. Failure to adequately match participants on past meditation experience may complicate the interpretation of findings from longitudinal studies of meditation training, as more experienced practitioners may have higher levels of performance at pre-assessment and may be differentially affected by the training itself. Furthermore, without empirically delineating the type, quality, and meaning of estimates of lifetime hours of meditation practice, the utility of these measures in informing research investigations of meditation training remains uncertain.

An intriguing direction for future research may be to utilize alternative cognitive training regimes as active comparison conditions in studies of meditation training. Although task-specific training rarely leads to generalizable improvements in attention and executive control, some forms of cognitive training such as musical training, action video games, working memory training, and meditation practice have each emerged as potential paradigms shown to impact general cognitive processes not explicitly trained during practice (Green and Bavelier, 2008; Jaeggi et al., 2008; Bavelier and Davidson, 2013). It will be important for researchers to begin to compare the effectiveness of different training regimes in improving attention and meta-cognitive awareness. Furthermore, the inclusion of wait-list and active control comparison conditions in longitudinal studies of meditation may be useful in examining the unique or overlapping effects of meditation practice while experimentally controlling non-specific factors such as the training environment.

Taken together, our findings support the view that Vipassana meditation training facilitates the efficient management of attentional resources and is accompanied by experiential changes in feelings of attentional stability and clarity that correspond to measureable changes in sensorimotor performance (RT variability). Further, these attentional benefits occur without concomitant increases in self-perceived effort or motivation. It will be useful for future studies to further explore the utility of meditative introspection as a means of refining phenomenological investigations of cognition (Lutz et al., 2002; Lutz and Thompson, 2003). Combining phenomenological, behavioral, and electrophysiological approaches is crucial to advancing our understanding of the brain mechanisms underlying attentional stability and the impact of meditative training on cognition. The present study underscores the importance of examining felt experience in order to advance our understanding of the phenomenological correlates of cognitive processes, and in particular, change resulting from intensive mental training.

### Conflict of interest statement

The authors declare that the research was conducted in the absence of any commercial or financial relationships that could be construed as a potential conflict of interest.

## Footnotes

^1^Demographic variables, including participant age, estimated hours of lifetime meditation experience, and years of experience, were also examined in this model, but were not significant predictors of A' or RT CV (all ps > 0.05) and were excluded from this and subsequent analyses.
